# Supplementary material for: History of breastfeeding but not mode of delivery shapes the gut microbiome in childhood
Source: PLoS One. 2020 Jul 2;15(7):e0235223. doi: 10.1371/journal.pone.0235223 (PMC7332026; doi:10.1371/journal.pone.0235223)
Supplement: Attachment — Submitted filename: Response to ReviewersR2.docx [file pone.0235223.s003.docx]

Dear Dr. Whisner,

Thank you for the opportunity to revise our manuscript entitled, “History of breastfeeding but not mode of delivery shapes the gut microbiome in childhood”. We have reviewed the comments raised by you and reviewers and have provided our responses along with a tracked-changes version of the manuscript.

Editor

1. *For children, the World Health Organization suggests that this approach to reporting child size and body weight status are more accurate given that they are in a period of more intense growth. This is a best practice in both clinical and research settings, and should therefore be carried out in this paper before it will be deemed acceptable for publication.*

We now report body size as body mass index as a percentile on line 122.

2. *The paper suggests that formula feeding is fine and may be beneficial given the promotion of gut maturation in Bacteroides. This message needs to be softened throughout the manuscript as many health agencies support breastfeeding as the gold standard for gut and systemic child health. Please revise the manuscript to ensure that formula feeding is not viewed as equal or superior to breastfeeding.*

We have softened the language throughout the manuscript to avoid suggesting that formula feeding may be equal or superior to breastfeeding, see lines 203, 240, and 305 in particular.

Reviewer #1:

No Comments

Reviewer #2:

1. *I read your rationale why you used BMI only. But it lacks clarity on the predominant body size of the children. Please use BMI% for the BMI of 20 to make sense to readers. I know it's age corrected but it still doesn't tell me of the child's weight status; normal (5% to 85%) over/under weight, (85%-<5th%) obese >95%.*

As noted above in the response to the Editor, we now report body size as body mass index as a percentile on line 122.

2. *The child's rearing environment is confusing, especially Table 1. Does the box 'sibling genetically related and adoptive home mean that of the 25 adopted children, there were 17 genetically sibs who were reared together in an adoptive home? The box 'child genetically unrelated to adopted child and biological home mean that in the biological home, 14 of children were there who were not related to the adoptees? For example, step-siblings and not half-siblings?*

The reviewer’s interpretation of Table 1 is accurate. We have added a note in the Table to clarify this for readers.

3. *For feeding practices, did you also measure if the adoptive parents used breast milk from a milk bank or from friends/family/community support breast milk networks? That practice is becoming more common and could have occurred in the younger children in your sample.*

Unfortunately, we did not measure if the adoptive parents used breast milk or from friends/family/community support breast milk networks. We have noted this limitation on line 286.

4. *Was the delivery mode measured by questions from adoptive or birth mother when records were not available?*

Delivery mode was measured by questions from adoptive or birth mother when records were not available. This information is provided on line 112.

5. *In the results section, was the 21% breastfed combined from both genetically related and non-genetically related sibs?*

The 21% was across all children in our study. We now provide clarification on line 147 by adding, “In our study’s subsample…”, and this percentage also appears in Table 2.

6. *Minor changes*

*On line 195, add 'gut' between human and microbiome.*

*Line 222 Mention also that bacteriodes can also cause serious infections if escape outside the gut, potentially leading to septicemia too.*

*Line 282 The percentage of breastfed varies by age of infant. Do you mean ever breastfed, exclusively breastfed, or breastfed to 6 months? More specificity needed.*

*Line 292 remove formula; it may be interpreted as 'better than breastfeeding' and that's not the approach supported by WHO or multiple health agencies.*

All minor changes noted by the reviewer have been made.

Thank you again for the opportunity to revise our manuscript.

Sincerely,

Camille C. Cioffi
